# Hospital discharge for stroke patients: Transitional Care is Brain

**DOI:** 10.1093/eurpub/ckac131.312

**Published:** 2022-10-25

**Authors:** AC Sgueglia, M Zeduri, M Rissone, P Bertuccio, A Cavallini, A Martignoni, A Muzzi, S Cutti, AG Ambrosio, A Odone

**Affiliations:** 1 Department of Public Health, Università degli Studi di Pavia, Pavia, Italy; 2 Cerebrovascular Department, IRCCS Mondino Foundation, Pavia, Italy; 3 Unit of Cardiac and Cerebrovascular Disease, Fondazione IRCCS Policlinico San Matteo, Pavia, Italy; 4 Medical Direction, Fondazione IRCCS Policlinico San Matteo, Pavia, Italy

## Abstract

**Background:**

Among cerebrovascular disease, stroke is a life-threatening neurological event and a main cause of serious long-term disability, with relevant healthcare and economic burden. Treatment of stroke is time dependent and organised integrated stroke care enables quick and effective responses to reduce stroke-related death and disability. This study aimed at evaluating the amount of hospital discharge to transitional care facilities for stroke patients to support integrated care models in the city of Pavia (Italy).

**Methods:**

In 2017 in Pavia, Fondazione IRCCS Policlinico San Matteo started a partnership with Fondazione Mondino to build a specific stroke pathway, becoming a leading centre for stroke treatment. We conducted a retrospective chart review (RCR) of patient-centred data to quantify the volume of discharge for stroke patients. Two trained public health residents reviewed medical records with stroke admission diagnosis during 2021, analysing onset (e.g., Emergency Room, other hospital, emergency network), ward, treatment and discharge types (e.g., home, death, transitional care facility).

**Results:**

Our RCR found 669 patients with a stroke diagnosis treated at San Matteo hospital in 2021, the vast majority of which were admitted to the neurology ward (375 patients, 56%). The recanalization rate was 32% (150 on 464 ischemic stroke patients). Regarding the discharge type, 299 patients (45%) were sent home, while 297 patients (44%) needed transfer to rehabilitation or long-term care facilities. About 8% (52 patients) of the overall sample died in hospital.

**Conclusions:**

Our analysis showed that, while most stroke patients were discharged and sent home, more than two-third need to be transferred to continue to get the right healthcare from the right professional. Transitional care facilities should receive the greatest consideration by systems and providers seeking to implement care models to reduce residual neurological disabilities for stroke patients.

**Key messages:**

• A fast and accessible emergency chain is essential to reduce residual neurological disabilities and the related healthcare and economic burden in stroke patients.

• Extending the stroke path model to other time-dependent diseases is increasingly high-priority to shape a strong and resilient healthcare system, ensuring qualified health coverage.

